# OP18 Incorporating Environmental Impacts In The Economic Evaluation Of Health Technology Assessment: State Of The Art And Challenges

**DOI:** 10.1017/S0266462324000813

**Published:** 2025-01-07

**Authors:** Carmen Guirado-Fuentes, Analía Abt Sacks, Lidia García-Pérez, Carme Carrion, Mar Trujillo-Martín

## Abstract

**Introduction:**

Environmental impact has been poorly addressed in health technology assessment (HTA) processes despite its potential role in promoting more sustainable health systems. Initiatives to incorporate this dimension into economic evaluations (EE) that support HTA are few and far between. We aim to identify the state of the art and challenges for incorporating environmental impact into the EE of HTA.

**Methods:**

We conducted a scoping review to identify publications on the assessment of the environmental dimension of health technologies from different approaches: establishment of theoretical frameworks and methods; data search strategies; identification of parameters, designs, and indicators; as well as descriptions of practical applications in HTA (literature review, EE, or budget impact analysis). The literature search was conducted through PubMed. Selected studies should provide insights to incorporate environmental impact into the EE of HTA regardless of the technology or environmental aspect considered (carbon footprint, use of resources, waste generation, etc.).

**Results:**

From a total of 219 references initially identified, 22 publications meeting the selection criteria were found. The holistic approach is recognized as the most appropriate for incorporating the environmental dimension, through the evaluation of the entire life cycle of the technology, as well as the management of the disease and the use of resources throughout the care process. A large amount of information and accurate estimates about the impact of the technology are needed. Therefore, the first reported approaches have focused on particular aspects of the environmental impact of a health technology (mainly the carbon footprint).

**Conclusions:**

The practical incorporation of the environmental dimension into the HTA is still very incipient. Foundations have begun to be established for its incorporation into economic evaluation. A consensus is required on the most appropriate methodologies and tools to collect the necessary data. It would also require a multidisciplinary approach and a framework for cooperation between all the stakeholders.

